# 2175. Emergence of Dual-Carbapenemase Producing Enterobacterales in Chile After the COVID-19 Pandemic

**DOI:** 10.1093/ofid/ofad500.1797

**Published:** 2023-11-27

**Authors:** Aura Villamil, Paula I Rodas, Ana M Quesille-Villalobos, Jose R W Martinez, Manuel Alcalde-Rico, Valeria E Quiroz, Lina M Rivas, María P Riquelme, Camila Solar, Araos Rafael, Lorena Dias, Patricia Garcia, José M Munita

**Affiliations:** Universidad del Desarrollo, Santiago de Chile, Region Metropolitana, Chile; Universidad del Desarrollo, Santiago de Chile, Region Metropolitana, Chile; Universidad del Desarrollo, Santiago de Chile, Region Metropolitana, Chile; Universidad del Desarrollo, Santiago de Chile, Region Metropolitana, Chile; Hospital Universitario Virgen Macarena, Santiago, Region Metropolitana, Chile; Universidad del Desarrollo, Santiago de Chile, Region Metropolitana, Chile; Universidad del Desarrollo, Santiago de Chile, Region Metropolitana, Chile; Universidad del Desarrollo, Santiago de Chile, Region Metropolitana, Chile; Universidad del Desarrollo, Santiago de Chile, Region Metropolitana, Chile; Universidad del Desarrollo, Santiago de Chile, Region Metropolitana, Chile; Univerdidad del Desarrollo, Santiago, Region Metropolitana, Chile; Pontificia Universidad Catolica de Chile, Santiago, Region Metropolitana, Chile; Clínica Alemana - Universidad del Desarrollo, Santiago, Chile

## Abstract

**Background:**

Carbapenem-resistant Enterobacterales (CRE) are a major public health threat, largely due to the presence of carbapenemases, which are globally disseminated in mobile genetic elements. The emergence of CRE carrying multiple carbapenemases has been reported in several countries, particularly after the COVID-19 pandemic. In this study, we report the emergence of dual-producing CRE (DP-CRE) in Chile and provide a phenotipic and genomic characterization using whole-genome sequencing (WGS).

**Methods:**

We evaluated the presence of carbapenemase in a total of 1367 CRE isolates recovered from invasive infections in 11 healthcare centers since 2018. Among them, 9 DP-CRE were detected and included in this report. Antimicrobial susceptibility was tested by broth microdilution and disk diffusion methods (CLSI, 2023), while *bla*_KPC_, *bla*_VIM_, *bla*_IMP_ and *bla*_NDM_ genes were detected by PCR. WGS was carried out using short and long reads (Illumina and Oxford Nanopore) and hybrid assemblies were performed.

**Results:**

All 9 DP-CRE identified were recovered between November 2021 and June 2022 from 3 healthcare centers of a single city. In terms of species, 6 were identified as *E. coli*, one isolate of *K. pneumoniae,* one *K. oxytoca* and one *C. freundii*. All DP-CRE identified carried the combination of *bla*_KPC_ and *bla*_NDM_. Genomic analyses confirmed all but one isolate carried *bla*_KPC-2_ and *bla*_NDM-7_. The remaining genome belonged to a *K. pneumoniae* that harboured *bla*_KPC-3_ and *bla*_NDM-7_. All 9 isolates exhibited resistance to all β-lactams, including carbapenems, aztreonam (ATM), cephalosporins and β-lactam/β-lactamase inhibators. Cefiderocol (FDC) was the only compound active against all the isolates. Also, all the DP-CRE became susceptible to ATM when combined with ceftazidime/avibactam (CZA). Hybrid assemblies revealed that *bla*_KPC_ and *bla*_NDM_ were harboured on independent plasmids (∼58,900 bp and ∼41,100 bp, respectively) as shown in Figure 1.

**Figure:**
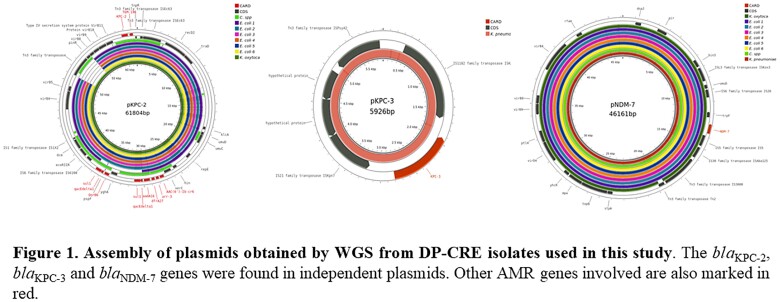

**Conclusion:**

To the best of our knowledge, this is the first report of the emergence of DP-CRE in Chile after COVID-19 pandemic. Our results highlight the relevance of active surveillance of multidrug-resistance pathogens. FDC and CZA/ATM were the only compounds that remained active *in vitro* against these pathogens.

**Disclosures:**

**All Authors**: No reported disclosures

